# Opioid-specific harm reduction in the emergency department: how staff provide harm reduction and contextual factors that impact their capacity to engage in harm reduction practice

**DOI:** 10.1186/s12954-024-01088-6

**Published:** 2024-09-18

**Authors:** Sunny Jiao, Vicky Bungay, Emily Jenkins, Marilou Gagnon

**Affiliations:** 1 School of Nursing, University of British Columbia, T201-2211 Wesbrook Mall, Vancouver, BC V6T 2B5 Canada; 2 School of Nursing, University of Victoria, 3800 Finnerty Road, HSD Building A402a, Victoria, BC, V8P 5C2 Canada

**Keywords:** Harm reduction, Emergency department, Acute care, Unregulated substance use, Complex adaptive systems, Nurses, Physicians, Case study, Mixed methods

## Abstract

**Background:**

Emergency Departments (ED) staff, including nurses and physicians, are most directly involved in the care of people who use unregulated substances, and are ideally positioned to provide harm reduction interventions. Conceptualizing the ED as a complex adaptive system, this paper examines how ED staff experience opioid-specific harm reduction provision and engage in harm reduction practice, including potential facilitators and barriers to engagement.

**Methods:**

Using a mixed methods approach, ED nurses and physicians completed a self-administered staff survey (*n* = 99) and one-on-one semi-structured interviews (*n* = 15). Five additional interviews were completed with clinical leaders. Survey data were analyzed to generate descriptive statistics and to compute scale scores. De-identified interview data were analyzed using a reflexive thematic analysis approach, which was informed by the theory of complex adaptive systems, as well as understandings of harm reduction as both a technical solution and a contextualized social practice. The final analysis involved mixed analysis through integrating both quantitative and qualitative data to generate overarching analytical themes.

**Results:**

Study findings illustrated that, within the context of the ED as a complex adaptive system, three interrelated contextual factors shape the capacity of staff to engage in harm reduction practice, and to implement the full range of opioid-specific harm reduction interventions available. These factors include opportunities to leverage benefits afforded by working collaboratively with colleagues, adequate preparation through receiving the necessary education and training, and support in helping patients establish connections for ongoing care.

**Conclusions:**

There is a need for harm reduction provision across all health and social care settings where people who use unregulated opioids access public sector services. In the context of the ED, attention to contextual factors including teamwork, preparedness, and connections is warranted to support that ED staff engage in harm reduction practice.

## Background

Harm reduction is a philosophy and a corresponding set of principles that inform programs, policies, and practices that aim to reduce the negative health, social, and legal impacts associated with drug use, drug policies, and drug law [1, 2]. Harm reduction as an approach has been taken up in health care in various ways, including take-home naloxone kits, safer use supplies, supervised consumption services, drug checking, safer supply prescribing, and opioid agonist treatment [3–10]. These interventions have demonstrated tremendous benefit, including reducing infectious disease transmission, overdose deaths, substance use practices that lead to harms; enhancing therapeutic relationships; and increasing referrals to community-based supports [11–14]. Despite these benefits, harm reduction interventions have not been fully implemented in the full range of health care settings where people who could benefit attend to care.

The Emergency Department (ED) is a particularly important setting where harm reduction interventions could provide crucial and timely support. It is well substantiated that barriers to appropriate and timely primary care can delay care seeking and/or make the ED the only source of possible health care [15, 16]. Such barriers include for example, inflexible community health schedules, transportation costs, fear of experiencing stigma, and the normalization of pain [17–19]. Previous studies in Canada have reported that people who use unregulated substances visit the ED at a rate that is 7 times that of a cohort matched by age, sex, region, and income quintile [20]. Furthermore, when people who use unregulated opioids present to the ED and are treated for non-fatal overdose, 5.5% die within one year of their visit, of whom, 20.5% die within the first month [21]. The ED is thus a critical point of connection, offering opportunities to deliver life-saving interventions, such as take-home naloxone kits, opioid agonist treatment, as well as referrals for ongoing care [22, 23].

The extant research concerning opioid-specific harm reduction in EDs has focused on two distinct interventions – take-home naloxone kits and opioid agonist treatment – offering descriptions of how these interventions are implemented, and contextual factors that may influence implementation [24–32]. Findings from these investigations focus on discrete elements of the ED, such as service provider knowledge, access to resources, and organizational policies, as influencing harm reduction implementation. However, there is growing evidence that attributing implementation challenges to parts of the system that are deemed “broken” without considering their various interrelationships [33] can lead to the formulation of strategies that produce unintended consequences and suboptimal outcomes [33, 34]. Counter to these approaches, a study by Jiao et al. [35] draws on central tenets of complexity theory that considers a systems approach to studying *how an ED is organized* to provide harm reduction. These findings illustrate that facilitators and barriers to implementation are shaped by interactions between policy and programming system elements (i.e., organizational policies that pertain to caring for people who use unregulated substances, the availability of substance use specialist services). Building upon these findings, this paper expands on these understandings to explore, through a complexity lens, how staff *engage in harm reduction practice in the ED*, including potential facilitators and barriers to engagement at the point of care.

As nurses and physicians are the primary groups providing care to people who use unregulated substances in EDs, and are ideally situated to implement opioid-specific harm reduction interventions in their practice [36–38], there is a need to understand how these two groups of providers experience harm reduction provision in the ED. In this study, we examined how nurses and physicians engage in opioid-specific harm reduction in the ED, as well as their perspectives about the factors that may influence their capacity to engage in harm reduction practice, including potential facilitators and barriers [25, 27, 28, 31, 32, 39, 40].

### Purpose statement and research questions

Positioning the ED as a complex adaptive system (CAS), the purpose of this paper is to examine how nurses and physicians engage in opioid-specific harm reduction in the ED, and to delineate contextual factors that may influence the capacity of staff to engage in harm reduction practice, culminating in the identification of facilitators and barriers. The specific research questions were: (1) In a CAS that is the ED, how do nurses and physicians as system agents engage in opioid-specific harm reduction provision? (2) What are provider-identified factors that may impact their capacity to engage in harm reduction practice?

### Emergency departments, harm reduction, and complexity theory

It is well substantiated that health care practice is situated within complex adaptive systems (CASs) that influence practice [41–44]. Within a CAS framework, systems such as the ED are considered whole entities, and structures and behaviours of the system are understood to emerge from characteristics of system agents (i.e., “diverse actors […] that interact with common and competing goals”) and their interactions [34, p. 630, 35, 45, 46]. Yet it is not possible to determine characteristics of the system by observing the properties of its constituent parts nor summing behaviours [45]. In recent years, there has been an increasing consensus that interventions that aim to tackle complex challenges within the health care system must cease to attribute challenges to implementation to specific parts of the system [33]. Instead, facilitators and barriers to implementation must be considered in light of how various elements of the system interact with one another [34].

Previously [35], we examined how an ED as a CAS is organized to provide opioid-specific harm reduction. We found that substance use specialists (also known as addiction medicine specialists) and non-specialists, as system agents, interacted in ways that enable harm reduction provision in the ED. However, limited access to specialist providers, in combination with specialist control, the reliance of non-specialists on specialist services, and safety concerns, created systemic tensions that hindered harm reduction provision. Contrary to this approach, previous investigations of contextual factors that impact the capacity of providers to engage in ED-based harm reduction practice do not adopt a complexity lens. Instead, they propose influential factors that place blame on certain parts of the system that are deemed “broken” [33], including those at the service provider level; at the administrative, infrastructural, and logistical levels; at the policy level; and at the levels of interdisciplinary and interagency support.

Factors reported to influence harm reduction practice at the *service provider* level include knowledge related to the evidence base for harm reduction, and training related to prescription, procedures for use, and implementation [27, 31, 40]. Factors at the *administrative* level include support from hospital administrators, a culture of acceptance for harm reduction interventions, and consistency with missions of the acute care institution [25, 39, 40]. Factors at the *infrastructural* level include targeted electronic health record alerts and pre-established order sets [32, 40], while factors at the *logistical* level include time constraints and competing demands [25, 27, 28, 31, 32, 40]. At the *policy* level, the availability of guidelines and protocols, service provider consultation in policy development, and ambiguity with identifying eligible patients, influences harm reduction practices [27, 40]. Lastly, factors at the level of *interdisciplinary support* include the availability of physician champions and mentorship, clearly delineated roles and responsibilities, and allied health support [31, 32, 39, 40], while factors at the level of *interagency support* include timely access to community based services, and effective mechanisms for transitioning care to the community [28, 31, 32, 39].

In the present study, we drew on central tenets of complexity theory to situate our examination into how ED nurses and physicians engage in harm reduction practice, including their perspectives on potential facilitators and barriers. Findings of this study have the potential to improve providers’ ability to engage in ED-based harm reduction practice, and to support the effective implementation of harm reduction interventions in EDs and other similar care settings.

## Methods

### Research design

This paper draws on staff survey and interview data collected as part of the first author’s dissertation work, which examined implementation of harm reduction interventions for unregulated opioid use in an ED in Western Canada, and associated barriers and facilitators for implementation. The study employed a case study design, and was informed by theoretical tenets of complex adaptive systems [45]. Please refer to Jiao et al. [35] for further details.

### Setting

The study case was the ED at a large hospital located in Western Canada; an urban hospital that serves a patient population disproportionately impacted by health and social inequities. The hospital is known internationally for its providers’ expertise in harm reduction, including the implementation of various harm reduction interventions, the development of comprehensive harm reduction policies, and the availability of a variety of substance use specialist services, including Addiction Medicine Physicians, Addiction Assessment Nurses, the Rapid Access Addiction Clinic (RAAC), as well as an in-house Overdose Prevention Site, the full details of which are published elsewhere [35]. The hospital’s ED is one of the busiest EDs in the province and provides services to a large proportion of people who use unregulated substances and experience related health and social inequities [47]. There were more than 84,000 visits to the hospital’s ED in 2015–2016 [48].

### Data collection

Data collection included an online, self-administered staff survey (*n* = 99) and one-on-one semi structured interviews (*n* = 20) conducted by the first author. Staff surveys took place from April to July, 2021, and survey recruitment included the distribution of recruitment emails to ED staff by clinical leadership, and the placement of recruitment posters in the ED staff room. The 66-item survey included research team generated items to capture demographic characteristics, work and educational experiences, and current harm reduction practices as applied to opioid use. The survey also included the 22-item Drug and Drug Problems Perceptions Questionnaire (DDPPQ), which was developed to measure care providers’ attitudes towards patients using unregulated substances [49]. The scale consists of six subscales: Role adequacy; Role legitimacy; Role support; Task specific self-esteem; Work satisfaction; and Motivation [49]. It uses a 7-point Likert response ranging from “Strongly agree” to “Strongly disagree” [49]. Previous studies provide estimates of the reliability and validity of the DDPPQ, with a Cronbach’s α of 0.92 for the scale as a whole [50], and Cronbach’s α ranging from 0.734 to 0.955 for the various subscales [51, 52]. For the purpose of this study, we adapted the wording and language of the DDPPQ to minimize stigma and to reflect current understandings of substance use (such as changing the language of “drug user” to “people who use unregulated substances,” and “drug problems” to “harms associated with unregulated drug use”). We calculated Cronbach’s α for the study sample to be 0.857 for the scale as a whole.

Staff interviews took place from May to June, 2021. Participants were recruited via a question included in the staff survey which asked if the person would be interested in an in-depth interview to further discuss harm reduction implementation in the ED. These interviews were semi-structured, and focused on how agents of the system interact with one another to support opioid-specific harm reduction implementation, as well as facilitators and barriers to implementation in light of these interactions. The interview guide was informed by contextual factors deemed to influence harm reduction in the ED as identified by a review of the existing literature, and allowed for the capturing of detailed knowledge pertaining to items of the staff survey. As data were collected, the need for key informant interviews with clinical leaders as agents influencing harm reduction implementation was identified. Thus, 5 additional interviews were completed with clinical leaders responsible for harm reduction implementation within the health authority. Interviews with leadership took place in November, 2021 and ranged from 55 to 80 min in length. Recruitment for interviews with leadership was in the form of personal email communications. These interviews focused on leaders’ perspectives on policy, programming, and implementation of opioid-specific harm reduction interventions. Full details of interviews conducted are published elsewhere [35]. All interviews were audio recorded after obtaining permission from the participant and subsequently transcribed.

The two strands of data (staff surveys and interviews) were collected concurrently. Approval for this research was obtained from the University of British Columbia (UBC) Behavioural Research Ethics Board (BREB), Certificate of Approval no. H19-02470.

### Data analysis

Staff survey data were uploaded to SPSS™ 27 for analysis. Descriptive statistics (e.g., frequencies) were generated for specific variables associated with participants’ demographics and work experience characteristics. Frequencies were also generated for variables associated with the types of beliefs that staff held about people who use unregulated substances, staff perceptions of the availability of opioid-specific harm reduction interventions in the ED, factors staff perceive to influence ED harm reduction implementation, and education staff obtained through school and the workplace. SPSS™ was also used to calculate DDPPQ total scores, which were calculated by summating the Likert response for each scale item, with lower scores denoting positive attitudes towards people who use unregulated substances and vice versa [49].

Once the survey data was analysed, de-identified interview data were uploaded to NVivo™ 12 for review and analysis. For this analysis, we utilized reflexive thematic analysis [53, 54], and drew upon the theoretical construct of complex adaptive systems [34, 45], which is concerned with the emergence of system structures and behaviours as a function of patterns of interaction between agents of the system [34, 35]. We also adopted understandings of harm reduction as both a technical solution and as a contextualized social practice as proposed by Jiao [55]. As per Jiao [55], harm reduction as a technical solution refers to “interventions that decontextualized and have the primary purpose of facilitating behavioural changes that are deemed necessary to reduce the harms associated with [unregulated substance] use” (pp. 1–2), encompassing interventions such as take-home naloxone kits and opioid agonist treatment. Harm reduction as a *social practice*, on the other hand, refers to “interventions that are nuanced and situated within the contextualized […] lifeworld of PWUD [people who use drugs]” [55, p. 2]. These interventions consider and attend to the impact of broader, socio-structural factors (i.e., poverty, housing insecurity, etc.) on harms experienced related to unregulated substance use, and people’s capacity to manage and mitigate those harms.

As the theory of complex adaptive systems directs a focus on patterns of interaction between system agents [34, 35], we generated a thematic coding scheme that reflected staff’s experiences of providing harm reduction in the ED, and the types of contextual factors that impact their capacity to interact with colleagues and community-based service providers to carry out their roles related to harm reduction provision.

The final analysis involved integrating both quantitative and qualitative data to generate overarching analytical themes, thereby engaging in mixed analysis [56]. Mixed analysis offers the benefits of triangulation (i.e., the ability to compare qualitative findings with quantitative results) and complementarity (i.e., the ability to seek elaboration, illustration, enhancement, and clarification of findings generated via one data source with results generated via another data source) [57]. In conducting mixed analysis, we gave the two data sources approximately equal priority, and made mixed analysis decisions iteratively [56]. In determining analytical themes for the mixed analysis, we asked questions such as: In the CAS that is the ED, how do nurses and physicians engage in harm reduction provision? What are staff-identified factors that may impact their capacity to engage in harm reduction practice? What contextual factors impacted the capacity of staff to meet patient needs related to harm reduction?

## Results

The analysis of the study site’s ED as a case for harm reduction implementation illustrated there were an array of influential factors within the system that facilitate or hinder the capacity of nursing and physician staff to implement the full range of opioid-specific harm reduction interventions available in the ED and in the hospital more broadly. In the sections that follow, we first present the study’s participants (ED staff) and the context in which they provide care for people who use unregulated substances including harm reduction interventions. Following, we present the three interrelated analytic themes of *teamwork*, *preparedness*, and *connections* that illustrate the elements of the ED as a complex adaptive system that influence harm reduction implementation.

### Participants

A total of 99 participants completed the staff survey. Demographic and employment characteristics for the staff survey are detailed in Table 1. Overall, the sample included almost equivalent numbers of nurses and physicians, and most participants held regular employment staff positions. Experience levels varied with 55.6% (*n* = 55) of survey participants having worked in their respective profession (as a nurse or physician, not specific to the ED setting) for six or more years, and 49.5% (*n* = 49) of participants having worked in the ED setting for six or more years. Interviews were conducted with 15 staff (*n* = 5 physicians and *n* = 10 nurses) and ranged from 45 to 75 min in length.

**Table 1:** Staff survey: participant characteristics and length of work (*n* = 99)

Participant characteristic	*n* (%)
Self-identified gender (*n* = 98)	
Woman	61 (61.6)
Man	37 (37.4)
Age (*n =* 91)	
Mean (*SD*)	38.7 (10.9)
Range	25–67
Occupation (*n* = 98)	
Nurse	51 (52.0)
Physician	47 (48.0)
Employment status	
Regular ^a^	89 (89.9)
Casual ^b^	6 (6.1)
Temporary ^c^	4 (4.0)
Length of time in occupation	
$\:\le\:$ 5 years	44 (44.4)
$\:\ge\:$ 6 years	55 (55.6)
Length of time in ED practice	
$\:\le\:$ 5 years	50 (50.5)
$\:\ge\:$ 6 years	49 (49.5)
Length of time in current ED	
$\:\le\:$ 5 years	59 (59.6)
$\:\ge\:$ 6 years	40 (40.4)

^a^ “Regular” refers to permanent employment in a full-time or part-time position.

^b^ “Casual” refers to employment on an on-call basis without holding a full time or part time position.

^c^ “Temporary” refers to employment in a full-time or part-time position but only until the staff member who holds the permanent position returns from leave.

### Harm reduction in context

The study site’s ED has a longstanding history of providing care for patients who use unregulated substances. The hospital is home to the second in-hospital overdose prevention site in Canada, and has played, and continues to play, a tremendous role in responding to the ongoing drug toxicity crisis. Consequently, staff regularly care for people who use unregulated substances and who simultaneously experience multiple, intersecting structural inequities including poverty, housing insecurity, gender-based violence, and histories of trauma. As one nurse noted:

My experience with the population who uses drugs is basically on a daily basis. I would say about 75% of the population that I care for are people who use drugs of some sort. It is a huge part of job. – Emergency Nurse

In caring for people who use unregulated substances, ED staff provide numerous harm reduction interventions to support patient safety, encompassing interventions that embrace harm reduction as a technical solution and as a social practice [55]. As a *technical solution*, staff provide three specific harm reduction interventions at the level of direct patient care, including opioid agonist treatment (OAT) initiation, take-home naloxone kits, and safer use supplies (such as sterile needles and alcohol swabs). As a *social practice*, staff aim to establish a continuum of care to support that patients continue to engage with care upon their discharge from the ED and within their home settings and daily lives. To facilitate such, staff provide internal referrals to other hospital services as well as external referrals to community services (Table 2).

Several wraparound programs and services (i.e., services that are based outside of the ED but have a role in supporting ED care provision) make opioid-specific harm reduction implementation possible in the ED, and numerous organizational policies outline the types of interactions between system agents that are required to support implementation (full details are discussed elsewhere, see Jiao et al. [35]). However, the mere existence of these structures does not translate to harm reduction implementation in the ED. A necessary condition for implementation is that staff are *aware* of these interventions. For the most part, staff are aware of the types of harm reduction interventions that are available within their department. They are also cognizant of the option for referral to a variety of internal and external substance use and harm reduction services (Table 2).

**Table 2:** Staff perceptions of the availability of harm reduction interventions

Harm reduction intervention	*n* (%)
Provided by ED staff	
Opioid agonist treatment initiation (*n* = 96)	
Yes	85 (88.5)
No	7 (7.3)
Don’t know	4 (4.2)
Take-home naloxone kits (*n* = 96)	
Yes	90 (93.8)
No	3 (3.1)
Don’t know	3 (3.1)
Harm reduction supplies (*n* = 96)	
Yes	42 (43.8)
No	41 (42.7)
Don’t know	13 (13.5)
Internal referral	
Social work (*n* = 96)	
Yes	83 (86.5)
No	9 (9.4)
Don’t know	4 (4.2)
External referral	
Opioid agonist treatment services (*n* = 96)	
Yes	92 (95.8)
No	3 (3.1)
Don’t know	1 (1.0)
Harm reduction supply services (*n =* 95)	
Yes	78 (82.1)
No	5 (5.3)
Don’t know	12 (12.6)

In addition to *awareness* of existing harm reduction interventions, staff also acknowledged the impact of stigma and discrimination on the care experiences of people who use unregulated substances, including harm reduction provision. Many staff did not subscribe to prejudicial discourse about this patient population and held fairly positive attitudes about caring for people who use unregulated substances. Results of the staff survey illustrate that staff’s average total score on the DDPPQ was 61.8 (Table 3). The distribution of DDPPQ total scores was asymmetric with a skew towards the lower end indicating positive attitudes. There exists, however, an extent of variation, ranging from extremely positive attitudes to comparatively more negative attitudes. Staff were also cognizant that stigma and discrimination are fundamental to the experience of people who use unregulated substances when accessing health care, and that these experiences can have profound implications for attendance to care (Table 4). These beliefs were echoed in interviews with staff:

I think the biggest thing for people of that population [people who use unregulated substances] is that they just feel like they can’t access healthcare the same way that people who don’t use, do… They felt disrespected, they felt judged… so that is a huge barrier for people. – Emergency Nurse

**Table 3:** Results from the Drug and Drug Problems Perceptions Questionnaire (DDPPQ) (*n* = 99)

Variable	Mean (SD)	Range
DDPPQ total score (*n* = 94)	61.8 (15.2)	25–100
**Individual scale items of the DDPPQ**		
When working with people who use unregulated substances, if I felt the need to, I could easily find someone with whom I could discuss any personal difficulties that I might encounter. (*n* = 98)	3.07 (1.501)	1–7
If I felt the need I could easily find someone who would be able to help me formulate the best approach to a person who uses unregulated substances. (*n* = 98)	2.86 (1.478)	1–7
I feel I have a working knowledge of unregulated substances and the harms that are associated with their use.	1.90 (0.953)	1–5
I feel I know enough about the causes of harms that are associated with unregulated substance use to carry out my role when working with people who use these substances.	2.03 (1.044)	1–7
I feel I know enough about the physical effects of unregulated substance use to carry out my role when working with people who use these substances.	2.12 (1.013)	1–5
I feel I know enough about the psychological effects of unregulated substances to carry out my role when working with people who use these substances.	2.38 (1.131)	1–7
I feel I know enough about factors which put people at risk of developing harms that are associated with unregulated substance use to carry out my role when working with these individuals.	2.35 (1.072)	1–6
I feel I know how to counsel people who use unregulated substances over the long term.	3.83 (1.340)	1–7
I feel I can appropriately advise my patients about unregulated substances and their effects.	3.08 (1.209)	1–6

Note. For each item of the DDPPQ, respondents were asked to rate their level of agreement with a statement about caring for people who use unregulated substances. There were seven possible responses to each item on a scale of “Strongly agree” (which as given a score of 1) to “Strongly disagree” (which was given a score of 7). Item-specific mean scores and standard deviations were calculated using these numerical scores, where a score of 4 represented a neutral attitude, and a score of less than or more than 4 represented positive and negative attitudes respectively

**Table 4:** Beliefs about people who use unregulated substances (*n* = 99)

Variable	*n* (%)
They are among the most frequent attendees of EDs	
Strongly agree / agree	79 (79.8)
Neutral	11 (11.1)
Disagree / strongly disagree	9 (9.1)
They have complex social and health needs	
Strongly agree / agree	99 (100.0)
There is an inadequacy of community-based public sector services for this particular group	
Strongly agree / agree	73 (73.7)
Neutral	10 (10.1)
Disagree / strongly disagree	16 (16.2)
They experience significant stigma and discrimination in the health care system, in hospitals, and in EDs	
Strongly agree / agree	75 (75.8)
Neutral	14 (14.1)
Disagree / strongly disagree	10 (10.1)
These experiences of stigma and discrimination can impact their access and attendance to care (*n* = 98)	
Strongly agree / agree	88 (89.8)
Neutral	6 (6.1)
Disagree / strongly disagree	4 (4.1)

Working in an institution that provides care for people who use unregulated substances on a daily basis, ED staff are committed to care provision for this patient population, and have a desire to provide harm reduction as a part of quality patient care. Staff are additionally knowledgeable about the types of harm reduction interventions available in their practice setting and cognizant of the existence and the impact of stigma and discrimination. Overall, staff feel positive about caring for this particular group of patients. In the ensuing analytic themes, we present the types of factors that staff experience as influencing their capacity to engage in harm reduction provision – *teamwork*, *preparedness*, and *connections*, and how these factors shape harm reduction provision in the ED, as well as their impact for the delivery of care.

### Theme 1: teamwork

*Teamwork*, defined as being able to draw on the knowledge, experience, and expertise of, and to work collaboratively with, inter and intradisciplinary colleagues to support care provision, was central to shaping harm reduction provision in the ED. Collaboration, consultation, role clarity, and the attitudes of team members towards caring for people who use unregulated substances were particularly important factors shaping harm reduction implementation (see Table 5).

**Table 5:** Factors perceived to influence harm reduction implementation

Variable	*n* (%)
Interdisciplinary collaboration and support (*n* = 95)	
Crucial / quite a bit	80 (84.2)
Somewhat	9 (9.5)
A little / not at all	6 (6.4)
Occupational role clarity (*n* = 96)	
Crucial / quite a bit	59 (61.5)
Somewhat	26 (27.1)
A little / not at all	11 (11.4)
Leadership support (*n* = 95)	
Crucial / quite a bit	74 (77.9)
Somewhat	15 (15.8)
A little / not at all	6 (6.3)
Culture of harm reduction acceptance (*n* = 95)	
Crucial / quite a bit	81 (85.2)
Somewhat	8 (8.4)
A little / not at all	6 (6.3)
Electronic medical record alerts (*n* = 94)	
Crucial / quite a bit	64 (68.1)
Somewhat	18 (19.1)
A little / not at all	12 (12.8)
Training in prescribing / administering opioid agonist treatment (*n* = 96)	
Crucial / quite a bit	72 (75.0)
Somewhat	17 (17.7)
A little / not at all	7 (7.3)
Training in using / teaching patients how to use a naloxone kit (*n* = 96)	
Crucial / quite a bit	60 (62.6)
Somewhat	23 (24.0)
A little / not at all	13 (13.6)
Training in using / teaching patients how to use harm reduction supplies (*n* = 96)	
Crucial / quite a bit	57 (59.4)
Somewhat	25 (26.0)
A little / not at all	14 (14.6)
Information on the evidence base for harm reduction (*n* = 96)	
Crucial / quite a bit	61 (63.5)
Somewhat	25 (26.0)
A little / not at all	10 (10.4)
Information on community-based service providers (*n* = 93)	
Crucial / quite a bit	65 (69.9)
Somewhat	21 (22.6)
A little / not at all	7 (7.6)
Policies around transferring care to the community (*n* = 94)	
Crucial / quite a bit	74 (78.7)
Somewhat	14 (14.9)
A little / not at all	6 (6.4)

Staff noted specifically, that teamwork can facilitate harm reduction provision by helping to improve intervention provision timeliness. Providing missed doses of opioid agonist treatment (OAT) for example, was defined as a “time-consuming” intervention because staff must first obtain confirmation about the patient’s dose and whether that treatment has been provided from system agents external to the ED – the person’s community dispensary. However, teamwork and collaboration among system agents who are internal to the ED were seen to improve the timeliness of OAT provision, as explained by an Emergency Physician below:

I would have the [in-house] pharmacist and the Addictions Nurse chase it up. So they would have to then contact the [patient’s community] pharmacy and find out whether the patient had received the dose. We can check on PharmaNet [provincial prescription medication tracking system], and then have some idea of it, but it is never completely sure on PharmaNet. Sometimes they’ll [the community pharmacy] reverse it if the patient hasn’t picked it up, but they don’t always do that. So it can be difficult to tell from that, so we need the corollary. – Emergency Physician

Teamwork also facilitated harm reduction provision in the ED by allowing system agents to draw on the knowledge, experience, and expertise of one another in arriving at the most appropriate approach to care inclusive of preventing and managing opioid withdrawal. Clinical policies note that staff can provide the patient with doses of opioid medications that are comparable to the amount of unregulated opioids they use in the community setting [58]. Staff, however, noted that they may be hesitant to provide this intervention as the doses required are often over and above the therapeutic range for people without histories of opioid use. In such cases, staff reported that inter-colleague consultation was effective to assist their comfort and confidence in providing sufficient opioid doses as part of withdrawal management. Staff also relayed that, overall, when they needed help in formulating the best approach to caring for people who use unregulated substances, they were able to rely on their colleagues for support (*n* = 98, *M* = 2.86, *SD* = 1.478) (Table 4). Below, an Emergency Nurse speaks to how system agents consult, and draw on the expertise of one another to facilitate withdrawal management:

I usually discuss it with another nurse every time. Even, because I’ve given the high dose hydromorphone, like 150 mg of IV hydromorphone that they give at [an outpatient clinic], I’ve given that before in the emerg and I’m not comfortable with it, but I’ll doublecheck it with the doctor and discuss it with another nurse and then I’ll give it. – Emergency Nurse

Lastly, teamwork among system agents can help to ensure that a harm reduction intervention is offered through leveraging shared responsibility. Within this ED, while certain interventions, such as the provision of OAT, can only be initiated by one type of system agent (physicians), other interventions, such as distribution of take-home naloxone kits, can be initiated by multiple types of system agents (physicians and nurses). In the case of the latter, such overlapping duties can serve as what staff described as a “safety net” to ensure that the intervention is offered to the patient; the assumption being that if one type of system agent does not offer the intervention, another will. One type of system agent can also prompt the other, as explained by ED staff below:

Physicians will order it [take-home naloxone kits], but we [nurses] don’t obviously need an order. We can just give it to them [the patient] but it’s nice to see that the physician has ordered it because they’ve acknowledged that they need it… and then it will definitely get done. I feel like it maybe gets missed if it is not ordered… so it’s nice if the physicians order it because it kind of prompts us too. – Emergency Nurse

In the same way that ED staff experienced teamwork to facilitate their engagement in harm reduction provision, they found *a lack of teamwork* to hinder harm reduction provision. This was especially evident when there was a muddying of roles between occupational groups, and when staff encountered attitudes and assumptions on the part of their colleagues that were incongruent with the harm reduction approach and discriminatory towards people who use unregulated substances.

Within the context of role clarity, more than 60% (*n* = 59) of survey participants reported that occupational role clarity impacted harm reduction implementation in the ED (Table 5). For the most part, system agents were acutely aware of their roles related to harm reduction. They also reported that, if needed, they could find a colleague who could help them clarify their professional responsibilities (*n* = 98, *M* = 2.88, *SD* = 1.494) (Table 4). Role clarity, however, quickly became muddled when there were overlapping duties between occupational groups. That is, despite the benefits of shared responsibility, staff reported adverse implications of shared responsibility in the form of the *passing of responsibility* from one occupational group to another, which they saw as contributing inconsistences in harm reduction practice. As noted by one physician:

I think it [the responsibility to offer naloxone kits] should be shared. But part of the problem is if you make it a shared responsibility and it is not one person’s responsible, then nobody does it. So maybe it’s maybe easier to have a designated person… I think it’s inconsistent because it’s not one person’s responsibility and so everyone assumes the other person is doing it. – Emergency Physician

Work culture concerning acceptance of harm reduction as an appropriate intervention was also relevant for harm reduction (see Table 5). In particular, negative attitudes held by some staff that were discriminatory towards people who use unregulated substances and negated harm reduction, had a profound impact on interdisciplinary collaboration and diminished the capacity of ED colleagues to provide harm reduction interventions. Assumptions that perpetuated stereotypes about the dishonesty of patients who use unregulated substances were particularly problematic as noted in the excerpt below:

They’re [the patient is] starting to go into withdrawal and they manage with high doses of hydromorphone and you ask the doctor… and they’ll be like “oh give them Tylenol or Advil.” You’re like “I get it” but also, I get the sense that, at least one of the doctors has said [they don’t] want us to perpetuate the system of people coming in for minor things when they can’t get the down [heroin] that they need on the street, and so now they’re coming in with other concerns in the hopes of getting hydromorphone. That was [the physician’s] concern. – Emergency Nurse

Overall, it was evident that *teamwork* was a significant factor that shaped ED harm reduction provision. While staff experienced teamwork to facilitate provision in various ways, such as through leveraging delegation and shared responsibility, and allowing staff to draw on the knowledge and experience of one another, a lack of teamwork also had profound implications, especially when roles were ambiguous between occupational groups, and when colleagues held attitudes or assumptions that were incongruent with the harm reduction approach.

### Theme 2: preparedness

Preparedness was a salient factor that influenced staff’s capacity to engage in harm reduction provision. Preparedness referred to having the knowledge and skills required to carry out their respective roles to their full scope of practice when implementing harm reduction. *Education* was a fundamental part of preparedness.

Overall, staff reported they did not feel prepared to provide harm reduction as part of their patient care: a situation influenced by a dearth of education to assume these roles (see Table 6). Almost 70% (*n* = 67) reported never receiving any education in their entry to practice schooling concerning care for patients using unregulated substances (Table 6). Consequently, the workplace was central hub for staff to obtain such education, with 70% (*n* = 68) reporting having received such education in the workplace. Harm reduction education was available through a variety of formats, including in-service education offered by nurse educators during work hours, online modules, and lectures from Addiction Medicine Physicians.

**Table 6:** Education received through formal schooling and the workplace

Variable	*n* (%)
Education received through school	
Caring for people who use unregulated substances (*n* = 96)	
Yes	29 (30.2)
No	53 (55.2)
Don’t know / remember	14 (14.6)
Prescribe or administer opioid agonist treatment (*n* = 29)	
Yes	21 (72.4)
No	7 (24.1)
Don’t know / remember	1 (3.4)
Use a naloxone kit (*n* = 29)	
Yes	18 (62.1)
No	10 (34.5)
Don’t know / remember	1 (3.4)
Engage in overdose prevention conversations (*n* = 29)	
Yes	18 (62.1)
No	7 (24.1)
Don’t know / remember	4 (13.8)
Use harm reduction supplies (*n* = 29)	
Yes	16 (55.2)
No	11 (37.9)
Don’t know / remember	2 (6.9)
Education received through the workplace	
Caring for people who use unregulated substances (*n* = 96)	
Yes	68 (70.8)
No	24 (25.0)
Don’t know / remember	4 (4.2)
Prescribe or administer opioid agonist treatment (*n* = 68)	
Yes	60 (88.2)
No	6 (8.8)
Don’t know / remember	2 (2.9)
Use a naloxone kit (*n* = 68)	
Yes	54 (79.4)
No	11 (16.2)
Don’t know / remember	3 (4.4)
Engage in overdose prevention conversations (*n* = 68)	
Yes	34 (50.0)
No	26 (38.2)
Don’t know / remember	8 (11.8)
Use harm reduction supplies (*n* = 68)	
Yes	35 (51.5)
No	26 (38.2)
Don’t know / remember	7 (10.3)

They [the department offered] did these little coffee things, it was pre-COVID and you could come and have a cup of coffee and they would go through these boards and teach you about how to do the COWS [Clinical Opiate Withdrawal Scale] and how to look for precipitated withdrawal and the whole process of learning and educating about Suboxone^®^ starts, and then there was an online Learning Hub. – Emergency Nurse

Ad hoc education also occurred when seeking guidance from agents specializing in addiction care, such as from Addiction Assessment Nurses as noted below:

If we need help figuring out what we’re going to do for induction, if we are doing a Suboxone^®^ induction, they [the Addictions Assessment Nurse] can certainly assist us with regards to the framework of that. They are also a good education piece for nursing and for physicians with regards to how much medication should we give this guy to bridge and that comfort level of giving that medication, so you have somebody else who is saying “yes, it is okay to give that.” – Emergency Physician

There was also inconsistency in the type of education received (Table 6). Opioid agonist treatment (88.2%, *n* = 60) was the most common type of education received, with less people receiving education on harm reduction supplies as a source of intervention (Table 6). Staff spoke at length about variations in education noting that inconsistency in the availability of workplace education greatly influenced the uptake of education and type received. Uptake was described as complex, noting that educational content and formats often failed to consider the complexity among system agents’ work schedules or availability, as noted below:

One thing that I was not able to attend due to scheduling problems was [physician] from the psychiatry department actually hosted three sessions for emergency department staff, which from my understanding were sessions to talk about work in the emergency department at [hospital name] and some of the challenges and to learn a little bit about trauma and all that kind of stuff. That was my general understanding, that it was kind of a safe place for people to gather and talk about some of their experiences and learn other’s experiences, or patient experiences. That was offered. I would have loved to go but I was unable to go. – Emergency Physician

Additionally, variation in financial incentivization and the optional versus mandatory nature of education were described as barriers to accessibility. As one nurse noted:

It’s not mandatory [Suboxone^®^ modules]. There is an online thing on Learning Hub [online platform for staff education]. It was incentivized previously where there was a draw or something if you completed the course, but it is voluntary. – Emergency Nurse

Staff further added that, when they did receive workplace harm reduction education, these opportunities were often limited to the point of “onboarding” (when staff were first hired for their job), and not consistently available on a regular or ongoing basis. Although they appreciate the educational material covered, and understand their roles related to harm reduction provision upon completion of the education, the concern about “forgetting” what was learned was frequently expressed.

Overall, staff saw a lack of education as *undermining their preparedness* and *limiting the scope of care provision* in the ED. The impact of insufficient education played out in unique ways associated with staff’s role as physicians or nurses. Physicians specifically spoke to how education gaps affected their comfort level in prescribing harm reduction treatments and the impact for patient care.

I’m not as comfortable with methadone induction in the emergency department. It is not something I’ve done or learned about before. I’ve read about how to do it, or in a clinic setting, but no. Often people I see come in after overdosing, pretty significant overdoses, so starting something that might make them more sleepy, or have respiratory depression and things like that, I think is a limiting factor as well. – Emergency Physician

Physicians also voiced that a lack of education affected their ability to arrive at *appropriate dosing* for harm reduction related medications, which, in turn, impacted their ability to meet the patient’s care needs. Determinations of the correct dosage proved to be especially difficult in light of complex patient needs, such as when the patient requires withdrawal management in addition to OAT and/or pain management.

I struggle, for instance, not just meeting their [the patient’s] needs in terms of withdrawal but then figuring out how to meet their needs in terms of withdrawal and then additionally provide them with proper pain control for whatever the new issue is that is bringing them. If somebody is on X amount of Kadian^®^ [a type of OAT], I have no idea how to even start treating the pain that they have for their terrible cellulitis or osteomyelitis or whatever it is that they have. I can’t even fathom what am I going to give you that is going to do anything on top of these doses that you’re getting, so I think that is really tricky as well. It is not because we don’t want to, it’s because [we] don’t know how to address it. – Emergency Physician

For nurses, the lack of educational opportunities impacted their ability to determine *when* to administer harm reduction treatments and to respond when treatment complications arose. For instance, uncertainty concerning use of the Clinical Opiate Withdrawal Scale (COWS) – a tool used to rate signs and symptoms of opioid withdrawal and to monitor symptoms over time was a serious concern. A COWS score is used to determine the severity of one’s withdrawal and can influence clinical decisions such as when to induce Suboxone^®^ (a type of OAT). Suboxone^®^ should only be induced when the patient is in withdrawal, otherwise the person risks experiencing precipitated withdrawal (i.e., withdrawal symptoms brought on by OAT). Below, Emergency Nurses speak to how a lack of education affected their ability to provide Suboxone^®^ at the most clinically appropriate time and to prevent and manage potential complications.

Some people still don’t really, they’re not super comfortable with administration [of Suboxone^®^] and they don’t know to take a COWS score prior and that the patient has to be at a certain point [of withdrawal to induce the treatment]. – Emergency Nurse

I think the biggest thing that we’re not great on is if we’re getting into precipitated withdrawal, what do we do as nurses. How do we make sure [patients do not experience precipitated withdrawal], you can do the best you can in terms of your scoring. – Emergency Nurse

Inconsistent and unavailable education also influenced nurses’ ability to administer “as needed” (PRN) medications, which consequently negatively affected their ability to meet the patient’s harm reduction care needs. Without education concerning the timing, dosing, and purpose of PRN methadone for example, patients are left to experience greater withdrawal and limited pain management [59]. Similarly, insufficient knowledge about the interrelationships between opioid dosage and patients’ tolerance associated with a history of unregulated opioid use, left nurses uncomfortable with PRN dosages and contributed to hesitancy to provide this important intervention.

They’ll [Emergency Nurse] be like “oh, the patient is in withdrawal” or like really uncomfortable and they had methadone, but then you’re like “oh, but they had three PRNs and they weren’t given over night,” but now it’s the morning shift and it is too late to do much with the PRNs because you know the bigger dose is coming. I remember an Addictions doctor had said to me, “we do 30 [mg] because that’s the basic safe starting dose and then you PRN it, so that if we come in tomorrow and we’ve used the PRNs, we know we can comfortably increase it to X, and to [continue with] PRNs until we raise it to the right amount.”... Oh, so it’s not just about a breakthrough medication, it is actually about trying to titrate the right amount. – Emergency Nurse

Ultimately, inconsistent and unavailable education further impacted the ability of nurses to advocate for patients’ harm reduction needs. Nurses explained that, with inadequate education, they were limited to passively passing information onto physicians, and may not be able to advocate fully on the patient’s behalf. Having received the corresponding education however, allowed them to have proactive discussions with physicians to arrive at the most appropriate care for the patient.

Like safe supply, it all started happening in the community and I feel like emerg was never really part of that and so it’s one of those things, I just don’t really know what to do. I hate those things… I don’t really know how much are people getting every day, how do they get it, where do they get it, is it a daily dispensed thing, do people ingest it, do they crush it and inject it, like I don’t know any of the stuff about it. I feel like I’m very sort of in the dark about how that goes, so it’s hard for me to really proactively be a good advocate and nurse, I guess, when it comes specifically to that kind of stuff, other than to just make sure the doctor knows what is going on. – Emergency Nurse

As a whole, staff of the ED described education as critical in being *prepared* to carry out their roles as related to harm reduction. However, many did not receive the corresponding education, either through entry to practice education, or through educational opportunities offered through their workplace. Although specific implications for practice varied for nurses and physicians due to differences in professional scope (i.e., prescription vs. administration) – what remained constant was the profound impact of unavailable and inconsistent education on staff’s capacity to carry out their roles as system agents and to meet their patient’s care needs.

### Theme 3: connections

In addition to teamwork and education as influential factors for staff’s capacity to carry out their roles when implementing harm reduction, another crucial consideration relates to support that staff receive to help patients make connections to community-based services and resources. ED staff identified that people who use unregulated substances have complex social and health needs (Table 3). Staff acknowledged the socio-structural context of people’s lives and viewed substance use as occurring against a backdrop of intersecting factors including chronic illness, mental health challenges, poverty, housing insecurity, racism, and trauma. Staff recognized the compounding nature of such factors and noted how, collectively, they contributed to a person’s unique social location in respect to their substance use and harms experienced.

Patients who struggle with addictions issues, usually they have pretty significant trauma histories or mental health issues, or they’re marginalized for other reasons, whether it be poverty or their ethnicity, or maybe generational trauma from being an Indigenous person in Canada.^1^ The patients are extraordinarily complex in many, many ways, and often we get a very small window into their lives, usually on their worst day, in the emergency department. – Emergency Physician

In recognizing the interrelated complexity between substance use harms and the structural disadvantages that exacerbate the potential for harms, staff embraced harm reduction as both a *technical solution* and a *social practice* [55]. Staff saw their role as not only resting at the level of *direct patient care*, but also as helping people *make valued connections* to available and appropriate community-based services and resources to support that people continue to engage with care. Staff saw *connection making* as essential to providing comprehensive harm reduction care, and recognized the limitations of solely offering interventions that subscribe to harm reduction as a technical solution.

This person [patient] wants to get off the drugs, he wants to get on Suboxone^®^, or methadone, but he has no place to live. I think that makes you press harder and get the social worker to focus on finding housing because we have an opportunity here but it’s going to fail if they don’t have housing, so “can you please help us find something?” – Emergency Physician

Overall, staff struggled to carry out their role in helping their patients make the necessary connections to community-based services and resources. Staff spoke about how, in order to facilitate such connections, they must manage multiple layers of systemic complexity and navigate a series of barriers – a situation influenced by intersecting issues of competing work demands, the limited availability of interdisciplinary support, as well as uncertainty related to available community-based services and resources.

The first barrier conveyed by staff relates to competing demands on their time and the resultant non-prioritization of connection making as a part of care delivery. The nature of the ED is such that nursing staff are simultaneously caring for four or more patients, while physicians may be involved in the care of up to 20 patients. As such, ED staff must constantly prioritize competing demands on their time. Notably, when caring for people who use unregulated substances, although nurses and physicians acknowledge the importance of helping to establish community-based connections, this aspect of care was not described as a *standard* part of their work, but rather, as something superfluous that staff *could* address if there was time. Many staff expressed concerns about the time required to engage with patients related to making community-based substance use and harm reduction connections, and reported that they may not have time in their workday to have a prolonged interaction with patients related to these needs.

I want to say in an ideal world that [helping the patient make community-based connections] should be part of our role. Some days you just don’t have time to go the bathroom, so it’s like anything extra might be not wanted. – Emergency Nurse.

Accepting that many ED staff do not consider connection making as a regular part of their work, yet striving to enact harm reduction as a social practice, the system creates workarounds through leveraging *interdisciplinarity* in helping patients make these valued connections. Interdisciplinary support is available through the Rapid Access Addiction Clinic (RAAC) – an outpatient clinic staffed by physicians, nurses, social workers, and peers. The clinic is located on hospital grounds and facilitates connections to an array of community-based supports – with the intention of offloading such responsibilities from ED service providers. Additionally, ED-based social work services are available to help patients connect to community-based supports, such as housing, income assistance, and outreach services (Table 2).

One of the main purposes RAAC was set up was to support the ED… There is such volumes and such high demand, a lot of the addictions patients that came into emerg… [The clinic was] set up to help decant emerg and help offload some of the pressure on the emerg department… I think it definitely alleviated a lot of the burden on the emerg department. – Clinical Leader

The staff were acutely aware of the availability of interdisciplinary support in enacting harm reduction as a social practice in the ED, and consistently drew on these supports. As noted in Table 2 for instance, over 80% (*n* = 78) of staff reported that the ED facilitates community-based connections for patients, and many spoke about how, instead of referring directly to community-based services and resources, they relied on referral to other agents of the acute care institution. Staff shared that oftentimes, they prefer to refer to the RAAC as opposed to ED-based social work services, whom they saw as overwhelmed and inundated with requests. Staff also believed that RAAC staff may be able to dedicate more time to addressing the patient’s interrelated and complex needs and can better attend to continuity of care.

I don’t refer to community supports. I refer to RAAC [the Rapid Access Addiction Clinic]. That is the umbrella under which people can access those types of services, or have that office liaise with community services in order to try to make things happen on that level… In terms of any kind of direct referral service from an emergency RN to a community-based support network, no, that it is not a thing. We refer to the resources available within [hospital name] and then from there, that would be their stepping stone towards accessing other outside services. – Emergency Nurse

In light of staff’s reliance on interdisciplinary support for connection making, the second barrier relates to the limited availability of such supports. The RAAC as a system agent and as interdisciplinary support was not available for the entire 24 period in which the ED operates. Afterhours (from 4 pm to 9 am), ED staff were left on their own with regards to making valued connections for patients – a capacity that solely depended on their *personal knowledge* of available and appropriate community-based supports. Staff described having very limited understanding of available and appropriate community-based services and resources or the mechanism for referral, contributing to the third and final barrier to connection making. Those who had some understanding noted that what they knew came from prior experience working as a care provider in the community context.

I am one of the people who doesn’t have a good understanding of just which community health centre or OAT provider would be appropriate for this patient. I don’t have a good map in my mind of what are the clinics. The only one I really know well is the Connections Clinic and that is from working at the [other workplace]… so I do direct people there, people who don’t have doctors. If they do have a doctor, if I happen to know their provider, I’ll direct them there, but if they have no doctor but they live Downtown Eastside, I’ll direct them to DCHC [Downtown Community Health Centre], or sometimes Three Bridges [Clinic], just because I know that they provide OAT. But I think not everybody knows that. – Emergency Physician.

## Discussion

Positioning ED care provision as situated within a complex adaptive system, this paper examines nurse and physician perspectives on the implementation of opioid-specific harm reduction in the ED, the types of factors staff experience as influencing their capacity to engage in harm reduction practice, and how these factors shape harm reduction in practice. Findings of this study illustrate that for ED staff to engage in harm reduction practice, and to carry out their roles as system agents related to harm reduction provision, they must be sufficiently prepared through receiving the appropriate education and training, be able to leverage the benefits afforded by working collaboratively with their colleagues, and be supported in helping patients make connections for ongoing care. Although existing studies have reported on the impact of education and training, and of interdisciplinary and interagency support on harm reduction practice in the ED, these studies do not adopt a systems perspective [28, 31, 32, 39, 40]. In fact, many such studies lack an overt statement of theoretical underpinnings [27, 28, 60], or draw on theoretical frameworks that attribute facilitators and barriers to implementation to a single element of the system [61]. A number of studies leverage theoretical frameworks that allow for the delineation of influential factors across multiple system elements [40, 62]. However, they do not attend to the emergence of system structures and behaviours as a function of patterns of interaction among agents of the system [34, 45]. To attribute implementation challenges to parts of the system without considering their interactions can lead implementation strategies that produce unintended consequences and suboptimal outcomes [33, 34]. In contrast, what this study offers is a careful consideration of interrelationships between parts of the system, leading to a comprehensive understanding of forces that affect change [63], and the formulation of effective and nuanced implementation strategies.

Through adopting a systems perspective, this study offers novel insights about contextual factors that impact the capacity of ED staff to engage in harm reduction practice – not only in light of not only how system agents *act*, but also how they *interact*. For instance, pertaining to the contextual factor of teamwork – which is concerned with how system agents work together – study findings underscore that while shared responsibility, and overlapping duties between different occupational groups, have the intended purpose of improving the consistency of which harm reduction interventions are offered, it can paradoxically contribute to inconsistencies in harm reduction practice. To this end, the existing literature has reported on the *passing of responsibility* between occupational groups including physicians, nurses, social workers, and pharmacists when responsibilities related to harm reduction provision in the ED were shared, to which staff provide a number of justifications – insufficient time in their work day, the presence of other occupational groups at the point of discharge, and inconsistencies with their scope of practice [27, 40]. Drainoni et al. [40] encapsulates the inherent irony of shared responsibility, and note that while shared responsibility has its merits – where everyone *can* provide harm reduction, it can also lead to scenarios where harm reduction is *nobody’s responsibility*. These findings, coupled with those of this study, suggest a need to concurrently leverage the benefits of shared responsibility while also being wary of its shortcomings. Hence, system agents must work together to ensure that shared responsibility translates to additional opportunities for harm reduction, and does not instead, serve as *justification for inaction*, nor lead to lost opportunities for harm reduction practice.

A second contextual factor influencing the capacity of staff to engage in harm reduction practice is consistent and available education. Findings of this study that many nurses and physicians did not receive substance use education within their entry to practice education is aligned with previous research. For example, in a survey of Canadian nursing students, 43% reported that they had received between 1 and 5 h of substance use education in their program to date, while for 20%, this subject was not broached at all [64]. Overall, students expressed that they do not feel prepared nor knowledgeable to provide care to people who use unregulated substances. Gagnon et al. [64] also found that although students report having been introduced to harm reduction as a philosophy of care, they may not be offered concrete ways of implementing harm reduction, nor learn the knowledge required to translate harm reduction into practice. Insufficient exposure to substance use education is similarly an issue for entry to practice medical school education [65–68]. A study by Wakeman et al. [69] notes that 27% of internal medicine residents report not receiving any substance use education in their entry to practice education, and 62% report feeling unprepared to provide treatment for substance use. Furthermore, the existing substance use education tends to focus on relaying biomedical knowledge, with limited attention to the practical skills and attitudes that are necessary in caring for patients in the clinical setting [70]. Given findings of this study, which elucidate how the oversight of education as an important contextual factor can have a myriad of detrimental consequences for harm reduction practice, it is of critical importance that substance use education is integrated into entry to practice nursing and medical education – a task that is decades long overdue [71]. These measures will help to ensure that nurses and physicians of the future will possess the practical knowledge and skills required to feel adequately prepared to engage in harm reduction practice to their full professional scope.

While research based in Ireland, the UK, and the Netherlands report on the inconsistent provision of substance use education at both “pre-qualifying” (prior to practice) and “post-qualifying” (after assuming practice) levels [72], the present study details the problem of inconsistent and unavailable workplace-based education pertaining to harm reduction in the Canadian context. Participants of the study identified various factors they believe to contribute to such inconsistencies, such as a lack of consideration for variation among staff work schedules, which appear to be novel. Furthermore, study findings bolster those of previous research that inconsistent and unavailable education has profound and grave implications for service provider preparedness in carrying out their roles as system agents as related to harm reduction provision, including impacts on staff’s ability to prescribe harm reduction related treatments and to determine when to administer such treatments [25, 28, 32, 62, 73, 74]. The impact of inconsistent and unavailable education on staff’s ability to administer PRN medications, and to advocate on behalf of the patient’s needs appear to be unique. Given such findings, it must be stressed that simply *offering* staff education is not enough. Instead, health care institutions must ensure that education for staff is consistently accessible so that staff are prepared to carry out their respective roles as system agents when implementing harm reduction.

Lastly, a third contextual factor that impacts the capacity of ED staff to engage in harm reduction practice is staff’s ability to facilitate connections with community-based services and resources. A key finding of this study is that ED providers did not see helping patients to establish community-based connections to be a *standard* part of their work, but as something extra they could address if they had time. This finding echoes existing literature which reports that ED staff found harm reduction, as a whole, to be *time-consuming* and i*nterruptive* to their workflow. This type of discourse was palpable across many harm reduction interventions, including take-home naloxone distribution and education [5, 25–27, 31, 40], opioid agonist treatment [32, 75], and withdrawal management [32]. Overall, staff felt that there was inadequate time in the clinical encounter for harm reduction provision due to the need to balance other clinical responsibilities. This type of discourse was particularly evident in the case of OAT as harm reduction intervention – where staff reported insufficient time for relationship building for the purpose of identifying eligible patients, to wait for patients to reach an appropriate clinical state for induction, and finally, for treatment induction and subsequent monitoring [28, 32]. To address these concerns, previous authors have recommended that ED based harm reduction be mindful of time pressures and competing priorities inherent in the ED [5]. It has also been proposed that EDs should employ dedicated staff to identify eligible patients for harm reduction, provide patient education, and establish outpatient follow up [28]. What is blatantly absent in these discussions, and urgently needed, is a critical examination as to *why* harm reduction, is not understood to be a *standard* part of ED practice, and why staff do not prioritize this facet of care – a topic worthy of investigation through future work in this field.

Finally, while the existing research identifies interagency support as a factor that impacts harm reduction practice in ED, this body of work tends to focus on the *insufficient capacity* of community-based resources. Winetsky et al. [74], for instance, report that community-based OAT providers are limited their capacity to accept patients who had their treatment initiated in the acute care setting, and this was a barrier to inpatient treatment induction. Previous research also highlights *issues with siloing* within the system, where care providers in the ED may face hurdles in referring patients in a timely manner due to disconnects between acute and community based care, as well as a lack of functioning referral mechanisms that can be accessed by ED staff [76]. Findings of this study, however, offer a different perspective and speak to interagency support in terms of *staff’s personal knowledge* of available and appropriate community-based resources – an issue that impacts staff’s ability to facilitate interrelationships and interactions with system agents external to the ED. We were not able to locate other studies that speak to a lack of knowledge related to community-based supports and their referral processes as a barrier to connection making, and, as such, these findings appear to be innovative. Future efforts to help patients make valued connections should attend to staff knowledge related to community-based supports – and consequently, staff’s ability to interact with other system agents to carry out their respective roles, in addition to considerations of insufficient program capacity and dysfunctional referral mechanisms [74, 76, 77].

### Limitations

The strengths of this study include its mixed methods approach, which allows for the integration of both quantitative and qualitative data in a mixed analysis, with the potential to generate rich, detailed, and nuanced findings. Additionally, the study draws on complexity theory to offer facilitators and barriers to engaging in harm reduction practice from the perspective of ED providers who are best positioned to implement harm reduction interventions in their practice. At the same time however, we must acknowledge a number of limitations to the present study. First, due to self-selection bias, we may be more likely to capture the perspectives of staff who are more accepting of harm reduction as an approach, and are more open to providing harm reduction interventions. Second, this study focuses on the perspectives of staff who are most directly involved in ED based harm reduction provision – nurses and physicians. However, there is a need for future research that examines factors that influence harm reduction practice from the perspective of a broader range of stakeholders, including Addiction Medicine Physicians, social workers, and security personnel. Lastly, although this study delves into staff perspectives of engaging in harm reduction practice in the context of a single ED, and presents highly contextualized and nuanced findings, we believe these findings may hold relevance for, and may be useful in, supporting harm reduction provision in other EDs and similar care settings.

## Conclusion

Given the many demonstrated benefits of harm reduction as an approach, there is a need for the implementation of harm reduction interventions across all health and social care settings where people who use unregulated substances attend to care. In the context of EDs, the perspectives of health care providers such as nurses and physicians are particularly relevant as these groups are most directly involved, and ideally positioned, to provide harm reduction in this setting. This study delves into ED nurses’ and physicians’ experiences of opioid-specific harm reduction provision, and identifies three interrelated factors as shaping staff’s ability to engage in harm reduction practice – *teamwork*, *preparedness*, and *connections*. Considerations related to these salient factors have the potential to inform and support current and future harm reduction implementation efforts in the context of EDs and across other health and social care settings.

## Data Availability

In accordance with consent agreed upon with participants, and to protect the confidentiality of ED staff and clinical leaders, the authors will not be sharing their full data set. Data supporting the reported results are available throughout the manuscript text in the form of participant quotations.

## Footnotes

1 1 We recognize that intergenerational trauma is not limited to Indigenous peoples. However, the unique features of colonization, inclusive of the destruction of family and heritage, are key structural drivers of intergenerational trauma for Indigenous peoples.

## Abbreviations

CAS: Complex adaptive system
COWS: Clinical Opiate Withdrawal Scale
DDPPQ: Drug and Drug Problems Perceptions Questionnaire
DTES: Downtown Eastside
ED: Emergency Department
PRN: Pro re nata (“as needed”)
RAAC: Rapid Access Addiction Clinic
